# Supplementary material for: Right Ventricular Myocardial Metabolism and Cardiorespiratory Testing in Patients with Idiopathic Pulmonary Arterial Hypertension
Source: Diagnostics (Basel). 2025 Oct 6;15(19):2523. doi: 10.3390/diagnostics15192523 (PMC12524233; doi:10.3390/diagnostics15192523)
Supplement: Supplementary file 1 [file diagnostics-15-02523-s001.zip › diagnostics-3808463-supplementary.pdf]

Table S1. Correlations between age and the main hemodynamics, cardiac MRI, PET/CT and CPET indices in the entire cohort (*n* = 34).

Parameter	*n*	Spearman, ϱ	t	*p*-Value
**Right heart catheterization**				
mPAP, mmHg	34	−0.008655	−0.048962	0.961254
PCWP, mmHg	34	0.315297	1.879457	0.069315
RAP, mmHg	34	0.055424	0.314006	0.755555
CI, L/min/m^2	34	−0.032308	−0.182854	0.856066
PVR, WU	34	−0.022437	−0.126953	0.899772
PAC, mmHg/mL	34	0.077930	0.442184	0.661330
**Cardiac MRI**				
RV ESV index, mL/m^2	34	0.176043	1.011650	0.319294
RV EF, %	34	−0.271689	−1.59698	0.120100
LV SV index, mL/m^2	34	−0.184003	−1,05896	0.297546
**PET/CT**				
[18F]-FDG SUVmax RV/LV lateral wall	34	0.050364	0.285262	0.777282
[13N]-NH3 SUVmax RV/LV lateral wall	34	−0.263606	−1.54585	0.131973
SUVmax 18F-FDG/SUVmax [13N]-NH3 RV/LV lateral wall	34	0.172522	0.990790	0.329221
**Cardiopulmonary exercise testing**				
VO_2 peak, mL/min/kg	34	−0.051987	−0.294481	0.770293
VO_2 peak Predicted, %	34	−0.178088	−1.02378	0.313616
VO_2/HR Predicted, %	34	0.090429	0.513648	0.611027
VE/VCO_2	34	−0.009495	−0.05371	0.957498

Footnote: CI, cardiac index; CPET, cardiopulmonary exercise test; ESVi, end–systolic volume index; EF, ejection fraction; LV, left ventricle; max, maximal; mPAP, mean pulmonary artery pressure; MRI, magnetic resonance imaging; PAC, pulmonary artery compliance; PCWP, pulmonary capillary wedge pressure; PET, positron emission tomography; PVR, pulmonary vascular resistance; RAP, right atrial pressure; RV, right ventricle; SV, stroke volume: SUV, standardized uptake value; [18F]-FDG, 18F-fluorodeoxyglucose; [13N]-NH3, ammonia; VE/VCO_2, minute ventilation per unit carbon dioxide production; VO_2/HR, oxygen pulse; VO_2 peak, peak oxygen consumption.
